# Desmoid fibromatosis post-cervical spine surgical intervention

**DOI:** 10.1093/bjrcr/uaae050

**Published:** 2024-12-17

**Authors:** Dana AlNuaimi, Shareefa Abdulghaffar, Reem AlKetbi, Badreya Khadim, Khalid Ibrahim

**Affiliations:** Department of Health, Abu Dhabi 20224, United Arab Emirates; Dubai Health, Dubai 1853, United Arab Emirates; Dubai Health, Dubai 1853, United Arab Emirates; Dubai Health, Dubai 1853, United Arab Emirates; Dubai Health, Dubai 1853, United Arab Emirates

**Keywords:** cervical spine, dysphagia, neck swelling, aggressive fibromatosis, desmoid tumour

## Abstract

Desmoid tumours are rare benign tumours that show locally aggressive and invasive features leading to potential complications. They can be quite challenging for the treating surgeon if they occur adjacent to neurovascular structures. The aetiology of these tumours is still unclear, but the incidence is higher in females and in patients with a history of trauma or surgical procedures, raising the possibility of genetic and hormonal factors as well as post-traumatic or post-operative inflammatory changes promoting the formation of desmoid fibromatosis. We report a case of a 34-year-old Asian female who presented to our hospital with a history of difficulty in swallowing due to an enlarging lump on the left side of her neck. Patient had a past medical history of previous cervical spinal disc surgery due to a herniated disc. Diagnostic imaging showed a large soft tissue mass centred between the left common carotid artery and the cervical vertebrae displacing the trachea and oesophagus to the right side. A biopsy was obtained, and histopathological examination was suggestive of a desmoid tumour. The patient underwent surgical resection of the tumour but returned later with recurrence and had to undergo the surgery again to achieve full cure.

## Introduction

Desmoid tumours, also called aggressive fibromatosis, are rare benign mesenchymal tumours encompassing only 3% of all soft tissue tumours and 0.03% of all neoplasms. Paraspinal desmoid tumours are even more rare, with only 15 cases reported in the literature up to the current date.^1^^,^^2^

They occur due to monoclonal neoplastic proliferation of fibroblasts or myofibroblasts within connective tissues and spread along fascia, aponeurosis, and muscular planes.

They do not metastasize; nevertheless, they are locally invasive and have a high recurrence rate post-resection of almost 50%. There is female predominance, being twice as common in females than in males, suggesting a hormonal response factor to its development.^1^^,^^2^

These tumours have varying rates of growth, and hormonal status seem to have an effect on the rate of growth.^3^ The disease peaks at 25-35 years of age, presenting in patients from 30 to 50 years old.^3^^,^^4^

## Case report

A 34-year-old female patient presented to the outpatient clinic complaining of difficulty in swallowing due to an enlarging mass on the left side of her neck for the past few months. The swelling was also associated with pain radiating to her left shoulder and mild shortness of breath.

She had no significant past medical history except for cervical spinal surgery due to a herniated disc 3 years ago with implant seen *in situ* at C5-C6 level. She has no significant family history or history of smoking or alcohol intake.

On examination, the patient was vitally stable. Chest was clear and abdomen was soft. Neurological examination was normal. An immobile, non-tender, and non-pulsatile swelling was noted on the left side of the neck.

Blood work-up showed normal blood cell count, electrolytes, renal, liver, and thyroid function tests. C-reactive protein was high measuring approximately 21.9 mg/L (normal value less than 5 mg/L). AFP-tumour marker was within normal limits.

**Figure 1.**
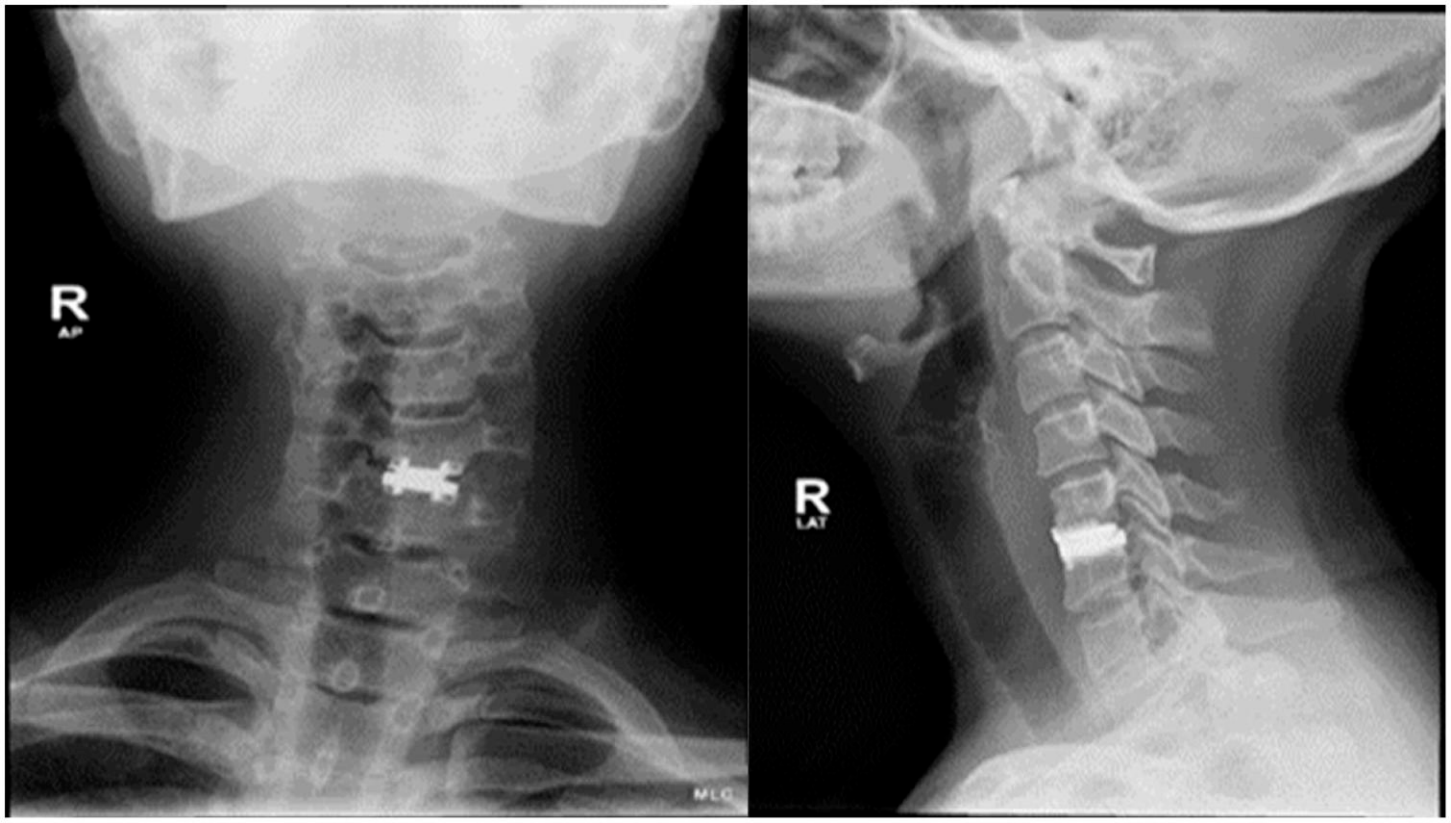
Cervical spine X-ray in the AP and lateral views showing an intervertebral disc implant at C5-C6 intervertebral space. Trachea appears slightly displaced to the right side.

Cervical spine radiographs were obtained and showed a maintained vertebral alignment with radio-opaque metallic disc spacer *in situ* at level C5-C6. The trachea appeared slightly displaced to the right side (Figure 1).

**Figure 2.**
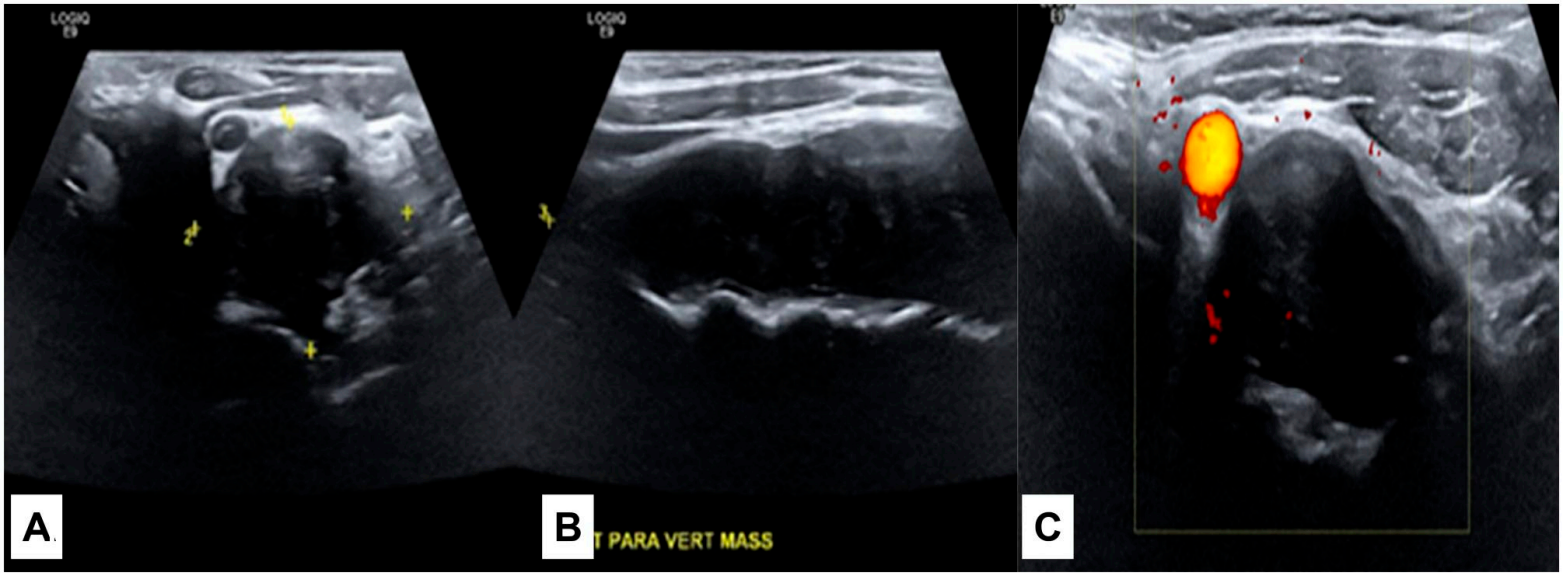
(A and B): Ultrasound of the neck of the left paraspinal region showing a hypoechoic heterogenous tubular soft tissue mass measuring approximately 30 × 34 × 80 mm in diameter. (C): Colour Doppler US image showing minimal vascularity.

Neck ultrasound showed normal appearance of the thyroid gland and no evidence of cervical lymphadenopathy. Nevertheless, upon scanning the left carotid region, a hypoechoic tubular and lobulated left paraspinal soft tissue mass centred between the left common carotid artery anteriorly and the cervical vertebra posteriorly measuring approximately 30 × 34 × 80 mm in diameter was seen, and it appeared to be displacing the left common carotid artery anteriorly and the trachea laterally. The mass showed a heterogeneous echotexture with minimal vascularity on colour Doppler images (Figure 2).

**Figure 3.**
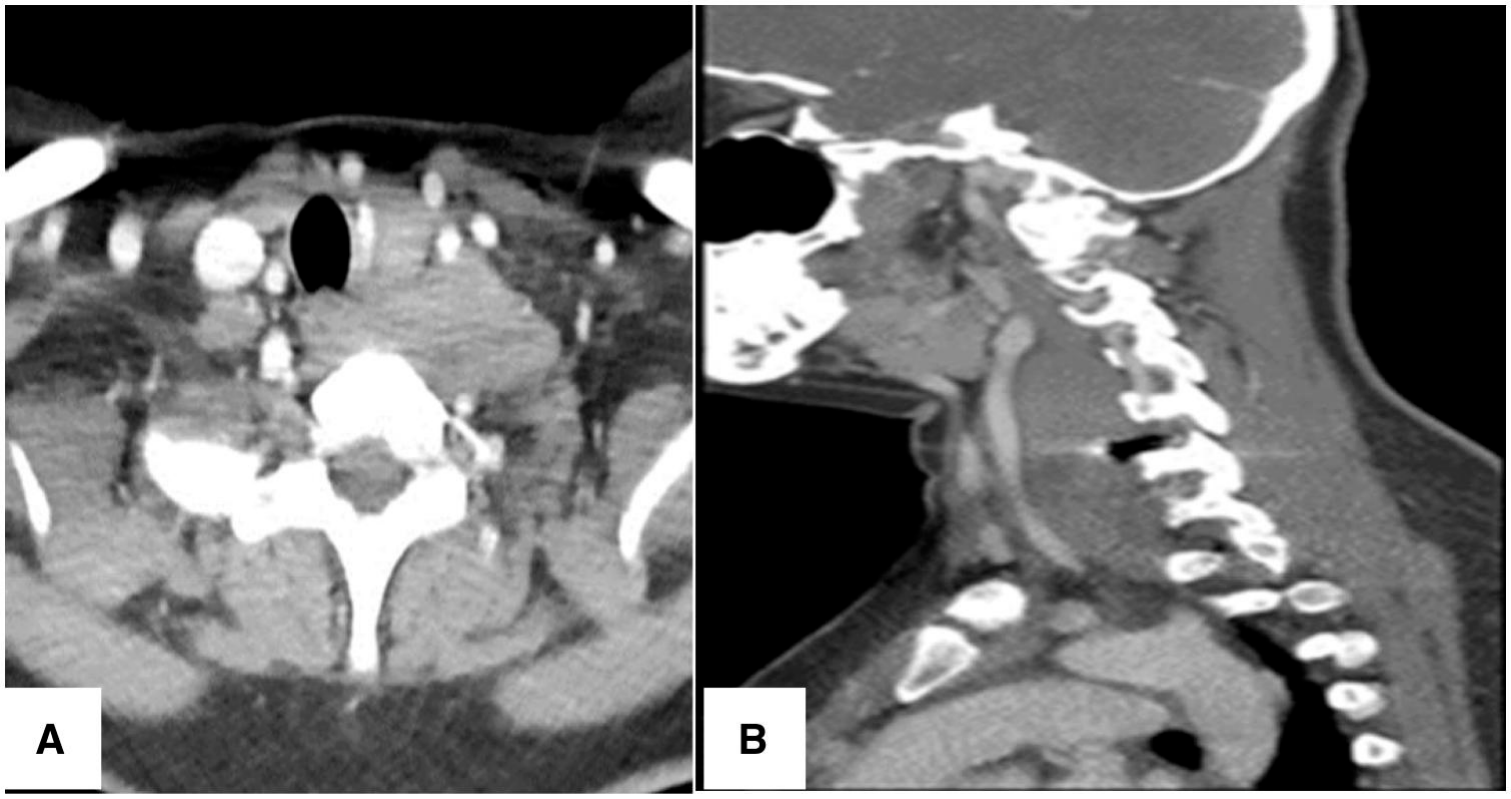
(A and B): Contrast-enhanced CT scan of the neck in the arterial phase in axial and sagittal sections showing a faintly enhancing iso-to-hypo-dense mass measuring approximately 85 × 32 × 26 mm in diameter displacing the left CCA anteromedially and the trachea and oesophagus to the right.

A contrast-enhanced CT scan of the neck showed a well-defined iso-to-hypo-dense mass compared to the surrounding musculature centred on the left carotid space extending from the bifurcation of the common carotid artery down to the thoracic inlet with minimal extension to the mid-line measuring approximately 85 × 32 × 26 mm in diameter. The mass was located between the common carotid artery and the internal jugular vein with displacement of the common carotid artery anteromedially. It showed homogeneous faint delayed enhancement at the late arterial/early venous phase exerting mass effect by displacing the trachea and oesophagus to the right side with no signs of local invasion. No calcifications were noted (Figure 3).

**Figure 4.**
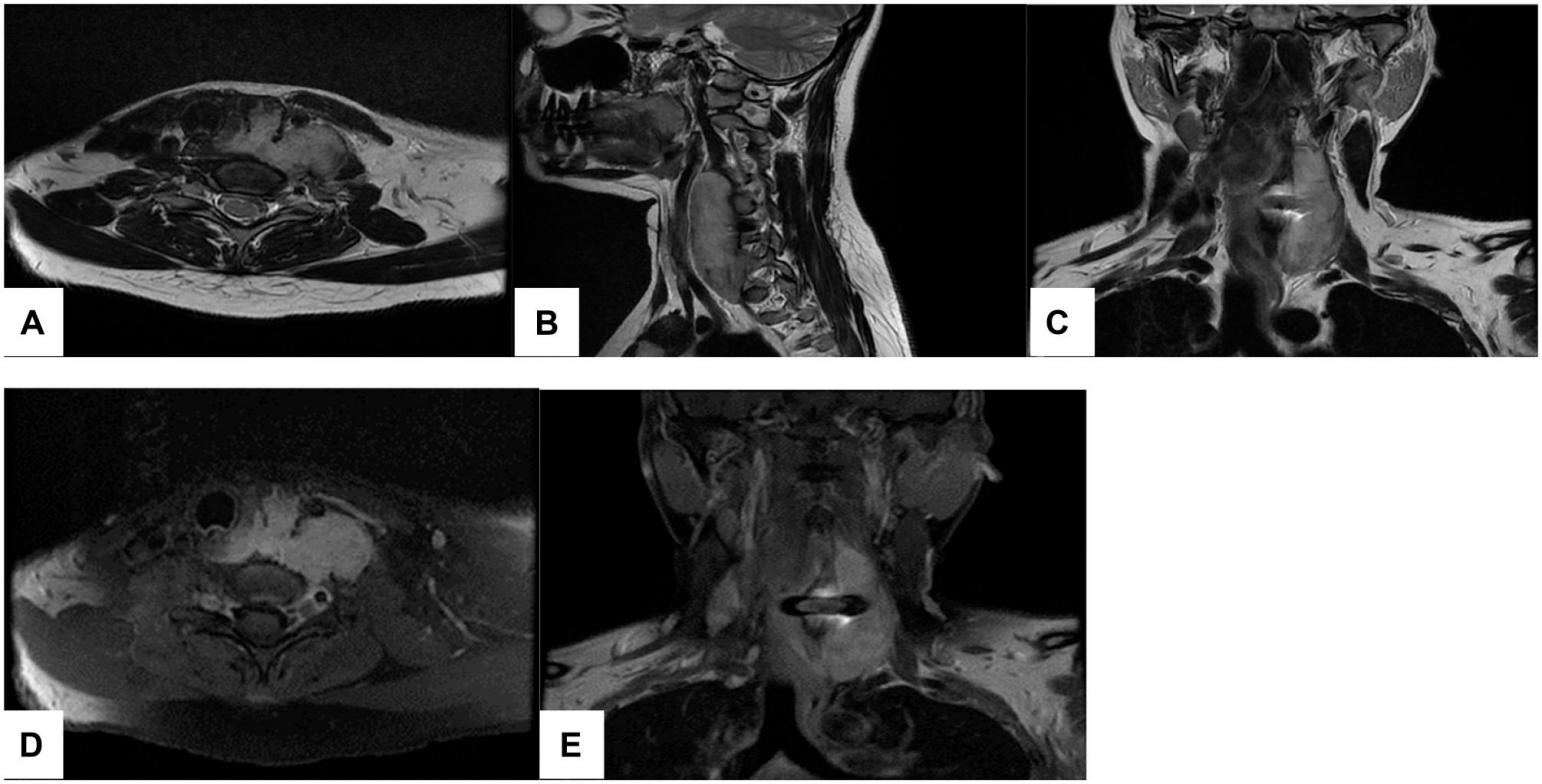
MRI of the neck. (A-C). Axial, sagittal, and coronal T2WI showing a hyperintense mass measuring approximately 85 × 32 × 26 mm in diameter displacing the left CCA anteromedially and the trachea and oesophagus to the right. (D and E): Gadolinium-enhanced axial and coronal T1WI showing homogenous enhancement of the mass.

Contrast-enhanced MRI of the neck showed a well-defined hyperintense lesion on T2WI centred in the left carotid space extending from the bifurcation of the common carotid artery downward to the thoracic inlet with minimal extension across the mid-line. It was centred between the left common carotid artery and the internal jugular vein displacing the common carotid artery anteromedially and showed enhancement upon contrast administration. It was seen exerting a mass effect and displacing the trachea and the oesophagus to the right side. A C5-C6 disc spacer was also noted showing a metallic artefact minimally indenting the thecal sac (Figure 4).

The differential diagnosis included neurogenic tumours such as schwannomas, conglomerated lymphadenopathy, carotid body tumours (paraganglioma), and other soft tissue tumours.

The patient underwent a core needle biopsy of the lesion, and histopathology found interlacing bundles of elongated spindle cells with wavy nuclei interspersed with collagen bundles; no atypia, pleomorphism, mitosis, or necrosis were seen. Immunochemistry was diffuse and strong for Beta-Catenin and Vimentin. Focal scattered positive for Smooth Muscle Actin (SMA), SOX 10, and S100 was observed. Findings were suggestive of fibromatosis.

The patient underwent mass excisional surgery in her home country, and the histopathology of the lesion also showed findings denoting aggressive fibromatosis. Unfortunately, after several months, the patient returned with recurrence of the lesion with the same symptoms of pain and neck swelling.

The C5-C6 intervertebral disc cage was removed with vertebral bodies osseous fusion done. The patient underwent another excisional surgery, which resulted in a complication of chronic hoarseness of voice likely due to the involvement of the recurrent laryngeal nerve.

Fortunately, she improved after the surgery with no recurrence of the lesion up to the current date.

## Discussion

The aetiology of desmoid tumours is still unclear. Post-operative inflammation at the surgical bed due to the presence of instrumentation may promote the formation of aggressive fibromatosis.^5^ They typically develop sporadically, most commonly seen in the abdomen, hip joints, and shoulder girdle.^4^^,^^5^ Previous history of trauma or surgical intervention increases its risk of development.^5^ Beta-Catenin signals are activated after bone fractures and during bone healing. Unregulated inflammatory cytokines were found to potentiate the growth of desmoid tumours, especially in radial and clavicular fractures.^5^

It is postulated that there is a mutation of Beta-Catenin occurring in the majority of reported sporadic cases. However, the risk is also higher in patients with genetic predisposition, such as in patients with familial adenomatous polyposis (FAP), where the FAP gene routinely regulates the degradation of Beta-Catenin. FAP occurs with mutation of the APC gene on chromosome 5q22.^1^^,^^4^ FAP, especially Gardner Syndrome variant, accounts for 10% of all desmoid tumours. These patients have an increased chance of 1000 times for developing desmoid tumours with an incidence of 5%-30%. A previous traumatic injury leads to local inflammation and up regulation of Beta-Catenin. This in turn leads to uncontrolled cellular proliferation of the monoclonal fibroblasts.^1^

Clinical features include a rapidly enlarging soft tissue mass and pain due to compression of adjacent structures.^2^^,^^3^ In the cervical spine region, a desmoid tumour may lead to deformity and pressure symptoms on vital organs.^5^ There is usually no or minimal neurological deficit at presentation. However, paraesthesia and limb paralysis may coincide with larger tumours.^5^^,^^6^

A desmoid tumour or aggressive fibromatosis must be considered by radiologists as a differential diagnosis of a soft tissue mass at any post-operative site.^4^^,^^5^

Desmoid tumours are classified according to their locations into intra or extra abdominal lesions.^2^ Another classification is superficial and deep fibromatosis.^4^ Superficial fibromatosis is typically smaller in size (less than 5 cm), slow growing, and does not involve deeper planes. On the contrary, deep fibromatosis is rapidly growing, infiltrative, and larger in size and has the tendency to recur after resection.^4^ Some desmoid tumours have a growth-arrest period, also called a plateau phase, where they stop increasing in size.^4^

The differential diagnosis of desmoid tumours includes fibromas, low-grade fibrosarcomas, neurofibromas, lymphomas, reactive fibroblastic proliferation, nodular fasciitis, solitary fibrous tumours, fibro-sarcomatous transformation, and metastasis.^3^^,^^6^

Diagnostic imaging plays an important role in imaging of these soft tissue lesions, in preoperative planning and evaluating post-surgical tumour recurrence or disease progression.^4^ On ultrasound, the mass usually appears lobular and infiltrative with a slightly hypoechoic echotexture.^4^

Computed tomography may show a non-specific soft tissue-enhancing mass, which is isodense to mildly hypodense to the surrounding musculature.^4^ Calcifications and central necrosis are not commonly seen in these lesions.^4^

Magnetic resonance imaging is the modality of choice as it provides superior soft tissue resolution and it can delineate the size of the mass and its surrounding structures. It shows a homogenous soft tissue mass, which is isointense in signal to muscles on T1WI and hyperintense on T2WI with low-signal fibrous bands on all sequences within the lesion and infiltrative margins. The mass shows moderate to avid enhancement upon Gadolinium contrast administration.^2^^,^^4^^,^^6^^,^^7^

Newer DCE-MRI enhances diagnosis specificity of these soft tissue tumours more than conventional MR imaging by using mathematical modelling for combining the representation of the tumour perfusion/leakiness (blood flow/blood volume and permeability) quantification as forward transfer constant (KTrans). The KTrans is characteristically high in malignant tumours, which show increased angiogenesis and micro-vascularity and would exhibit lower values in desmoid tumours, which are benign.^2^

Nevertheless, medical imaging cannot differentiate the mass from low-grade sarcomas and histopathology, and immunochemical analysis with core biopsy tissue sampling remains the gold standard in the diagnosis of desmoid tumours and shows interwoven spindle cells in bundles over a background of varying amounts of collagen matrix and sacrolemmic giant cells. They lack high mitotic counts often seen in malignant neoplasms.^2^^,^^3^^,^^7^

Immunochemistry is positive for Beta-Catenin, Desmin and SMA and may be positive for Vitementin and Estrogen Receptor Beta.^1^^,^^6^^,^^7^ Ki-67 proliferation index is usually lower than 1% in desmoid-type fibromatosis.^6^

A multidisciplinary team approach for the management of these tumours is important due to their proliferative nature, local infiltration, and at times, the proximity to important anatomical structures, as there is a possibility of a plateau-phase or even at times regression of the tumour.^1^^,^^5^

The management is routinely tailored to each individual case separately depending on the patient’s symptoms and anatomical location of the desmoid tumour and includes observation and surveillance for milder cases and wide en bloc resection aiming for clear margins for larger tumours.^1^^,^^8^ Adjuvant post-operative radiotherapy is prescribed if negative margins were not achieved during surgery or where surgery may result in further comorbidities, whilst studies have shown that local control after radiation therapy is 70%-80%.^6^^,^^7^ Studies have also shown a role for hormonal (anti-oestrogen) and anti-inflammatory therapy and new therapeutics such as immune inhibitors.^3^^,^^4^^,^^5^^,^^8^

Multiple surgical interventions are usually required for complete remission.^7^ Risk factors for recurrence after resection include the age of the patient, anatomical location, and size of the mass and regular-follow-up and monitoring is recommended.^1^^,^^2^ Recurrent desmoid tumours appear to be more aggressive in nature and are often difficult to differentiate from scar tissue.^3^

## Conclusion

Desmoid tumours are benign but have aggressive and invasive features, especially if they occur in proximity to neurovascular structures, and are extremely rare in the para-cervical region. We report a case of a 34-year-old Asian female who presented with history of difficulty in swallowing and an enlarging mass on the left side of her neck. The patient had a history of cervical spinal instrumentation. Radiology plays a major role in assessing desmoid tumours, and their differentials, including their size and proximity to important structures and enhancement patterns. Nevertheless, histopathology and immunochemistry are crucial for establishing the diagnosis. The patient underwent surgical resection twice due to recurrence and fortunately achieved no tumour recurrence up to the current date.

## Learning points

- Desmoid tumours are rare benign lesions of spindle cell origin that are locally aggressive but do not metastasize and must be considered as a differential diagnosis of a soft tissue mass occurring at post-operative sites.
- Diagnostic imaging plays a crucial role in delineating the size and anatomical boundaries of these tumours, especially to neovascular structures for preoperative planning and for post-surgical recurrence and disease progression. However, histopathology and immunochemistry remains the gold standard for establishing the diagnosis.
